# Development and Preliminary Psychometric Evaluation of a Questionnaire on Comprehensive Protection, Quality, and Humanization in Pediatric Hospital Care

**DOI:** 10.3390/pediatric18050137

**Published:** 2026-10-12

**Authors:** Germán Rojas Loyola, Begoña Polonio-López, Juan José Criado-Alvarez, Clara Rojas Melville

**Affiliations:** 1 Department of Pediatrics, “Nuestra Señora del Prado” General University Hospital, Integrated Care Management of Talavera de la Reina (SESCAM), 45600 Talavera de la Reina, Spain; 2 Faculty of Health Sciences, University of Castilla-La Mancha (UCLM), 45600 Talavera de la Reina, Spainjuanjose.criado@uclm.es (J.J.C.-A.); 3 Integrated Care Management of Talavera de la Reina (SESCAM), 45600 Talavera de la Reina, Spain; 4 Medical Admissions & Clinical Documentation Physician, Campo Añuelo Hospital, Extremadura Health Service (SES), 10300 Navalmoral de la Mata, Spain; claraerojasm@gmail.com

**Keywords:** psychometrics, reliability, validity, pediatrics, questionnaire, children’s rights, quality of pediatric care, humanization of care

## Abstract

Background/Objective: We aimed to develop a questionnaire for pediatric healthcare professionals assessing the rights of hospitalized children, service quality, clinical relationships, and care practices and to obtain preliminary evidence of its content validity, internal consistency, and exploratory psychometric properties. Methods: A four-phase methodological study was conducted: (0) instrument construction based on a review of the literature and relevant regulations; (1) expert content validation (*n* = 8) using the Content Validity Coefficient (CVC) and Kendall’s coefficient of concordance (W); (2) preliminary pilot testing (*n* = 10) for item refinement; and (3) post-pilot testing with pediatric healthcare professionals (*n* = 35) to examine internal consistency and exploratory psychometric properties. Descriptive statistics, the Kaiser–Meyer–Olkin (KMO) measure, Bartlett’s test of sphericity, principal component analysis (PCA) with Varimax rotation, average variance extracted (AVE), the heterotrait–monotrait ratio (HTMT), and correlations with a global satisfaction item (P50) were used. Results: The initial questionnaire comprised 77 items. Expert validation showed high CVC values for relevance (0.976), appropriateness (0.982), and clarity (0.973), with strong concordance among experts (Kendall’s W = 0.85, *p* < 0.001). During preliminary pilot testing, two single-choice questions were converted into two dichotomous items, yielding a 79-item post-pilot version. Refinement removed 18 items, resulting in a 61-item questionnaire. All four a priori theoretical dimensions showed Cronbach’s alpha values ≥ 0.70, providing preliminary evidence of internal consistency. Exploratory principal component analyses conducted separately within the four a priori theoretical dimensions yielded 4 to 6 components, accounting for 65.9–76.5% of the total variance. KMO values were modest (0.515–0.545), although Bartlett’s tests of sphericity were statistically significant. The retained items showed communalities and component loadings above 0.50. AVE and HTMT were calculated as exploratory indicators related to convergent and discriminant validity, respectively. The global satisfaction item (P50) loaded on the second component of Dimension 4 (communality = 0.60) and showed positive correlations with the remaining dimensions. Conclusions: The questionnaire showed high content-validity coefficients and acceptable preliminary internal consistency. The PCA, AVE, HTMT, and P50 findings provide exploratory information on item behavior and relationships among the theoretically defined dimensions but do not establish convergent, discriminant, criterion-related, or empirical dimensional validity. Given the small, single-center sample and modest KMO values, larger independent samples are required to confirm the dimensional structure and provide further validity evidence.

## 1. Introduction

Contemporary hospital management focuses on improving effectiveness and quality of care while reconciling better health outcomes with respect for human dignity [1]. Quality improvement is conceptualized as a systematic process aimed at implementing changes that strengthen patient safety, care coordination, and the satisfaction of both patients and healthcare professionals. The World Health Organization (WHO) promotes interventions that ensure health systems capable of delivering high-quality services grounded in adequate infrastructure, qualified personnel, and person-centered care [2,3].

Healthcare staff satisfaction, conceptualized as a positive emotional state derived from work experience and professional values, is an essential component of healthcare quality, requiring bidirectional communication channels that enable the identification of and response to organizational and clinical needs [4,5].

The humanization of care represents a fundamental pillar of the quality paradigm, although it extends beyond its boundaries [6]. In the hospital context, it entails a comprehensive approach to the individual across their biological, psychological, and social dimensions, underpinned by healthcare ethics centered on human dignity. This perspective, embedded in professional codes of ethics, aims to prevent a diminished view of professionalism that disconnects humanism from science [7]. From a bioethical perspective, humanization serves as the practical application of ethical principles in clinical practice, fostering respect, justice, and responsibility toward others [8]. Grounding humanization processes in bioethical principles safeguards the dignity, rights, and well-being of the individual [9].

The Doctrine of Comprehensive Protection is rooted in the Convention on the Rights of the Child (CRC), ratified by Spain and in force since 5 January 1991 [10,11], which promotes the protection of the fundamental rights of children and adolescents [12,13]. Similarly, the European Charter for Children in Hospital (1986) recognized 23 rights and established recommendations for healthcare staff, hospital authorities, and parents or guardians, applicable to all hospitalized children [14].

For these reasons, pediatric hospitalization demands welcoming and comprehensive environments that foster patients’ physical, emotional, and social well-being while respecting their rights to play, communicate, and learn. Hospitals implementing humanization strategies have demonstrated tangible benefits, including reduced stress and anxiety, shorter recovery times, reduced reliance on pharmacological interventions, and improved professional performance [15].

Within this conceptual framework, indicators have been proposed to evaluate the quality of hospital care for adolescents, incorporating dimensions of care experience (welcoming environment, physical setting, respect, trust, participation, and communication) and evidence-based care (psychosocial assessment, confidentiality, time alone, self-management and parental involvement, transition to adult services, educational support, and connections with support networks) [16].

In the emergency department, children and adolescents have unique needs that require specialized and differentiated care. Core values include empathy, physical separation between pediatric and adult areas, continuous training, the use of pediatric-specific tools, interdisciplinary collaboration, and clear communication with patients and families. Furthermore, the clinical expertise of pediatric care specialists is considered essential to identify and fulfill these needs [17]. Although these principles are particularly visible in emergency care, they are relevant to pediatric hospital care more broadly and are included here as an illustrative clinical context.

In this regard, pediatric healthcare personnel require robust ethical training to navigate the dilemmas inherent to clinical practice, avoiding paternalistic or dehumanizing attitudes [18,19]. Ineffective communication and the infringement of children’s rights are frequently the primary causes of dissatisfaction [20]. Moreover, pressure for administrative efficiency can lead to a reductionist view of the patient as a mere biological entity, neglecting their biopsychosocial dimension [21]. Dehumanization, consequently, constitutes a complex and multifactorial phenomenon [22].

Finally, organizational climate and institutional culture exert a decisive influence on healthcare quality. While climate reflects collective perceptions of the work environment, culture encompasses the underlying values and deep-seated meanings that sustain it [23,24]. Both are shaped by ethical deliberation, understood as a pathway for building a positive organizational culture [25].

Related instruments address overlapping aspects of care. Previous instruments have assessed individual or overlapping aspects of pediatric hospital care. Bisogni and colleagues assessed nurses’ perceptions of hospitalized children’s rights using 10 frequency-rated questions and two open-ended questions [26]. Child HCAHPS assesses parents’ or guardians’ experiences of pediatric inpatient care, including communication, safety, and comfort [27]. These instruments provide relevant points of comparison, although they differ in scope and respondent perspective. The overarching construct is healthcare professionals’ perceived quality of pediatric hospital care. Four a priori theoretical dimensions were defined to organize the content of the questionnaire: children’s rights, service quality, clinical relationships, and professionals’ own care practices. Clinical Relationship emphasizes communication and participation, whereas My Care Practice concerns the professional’s appraisal of their own conduct. WHO pediatric quality standards support attention to rights, communication, competent staff, and evidence-based care [28]. These dimensions were defined a priori on conceptual and content grounds and represent the intended organization of the instrument; they should not be interpreted as empirically established latent factors on the basis of the present study.

The design and validation of a questionnaire are essential to ensure valid and reliable measurements in the social and health sciences. Psychometric validation allows for the assessment of an instrument’s reliability, validity, and discriminative capacity, ensuring precise and actionable results [29]. Taken together, these interrelated dimensions support the need for a structured instrument that can assess them within a common framework and identify areas for improvement in pediatric hospital care. Recent evidence also highlights the value of healthcare professionals’ perceptions in evaluating the provision and quality of pediatric care. In a cross-sectional study conducted in two public hospitals in Greece, researchers examined healthcare professionals’ perceptions of emergency pediatric care provision, illustrating the relevance of professionals’ experiences and perspectives for identifying aspects of pediatric care that may require improvement [30]. However, assessing perceived quality of pediatric hospital care requires a broader conceptual approach that encompasses multiple dimensions of care rather than focusing exclusively on emergency care provision. In this context, the present study conceptualizes healthcare professionals’ perceived quality of pediatric hospital care as a multidimensional construct and contributes to its assessment through the development of a 61-item questionnaire designed to capture professionals’ perceptions across the main dimensions of pediatric hospital care. To date, however, the reviewed literature reveals no instrument that simultaneously integrates hospitalized children and adolescents’ rights, hospital service quality, the clinical relationship, and healthcare practice from the perspective of healthcare personnel. Consequently, this study aims to develop a questionnaire on comprehensive protection, quality, and humanization in pediatric hospital care and to obtain preliminary evidence regarding its content validity and internal consistency, while exploring item behavior and other preliminary psychometric characteristics. The four dimensions were specified a priori on theoretical and content grounds, whereas their empirical dimensional structure was not intended to be established in the present small-sample study.

## 2. Materials and Methods

**Figure 1:**
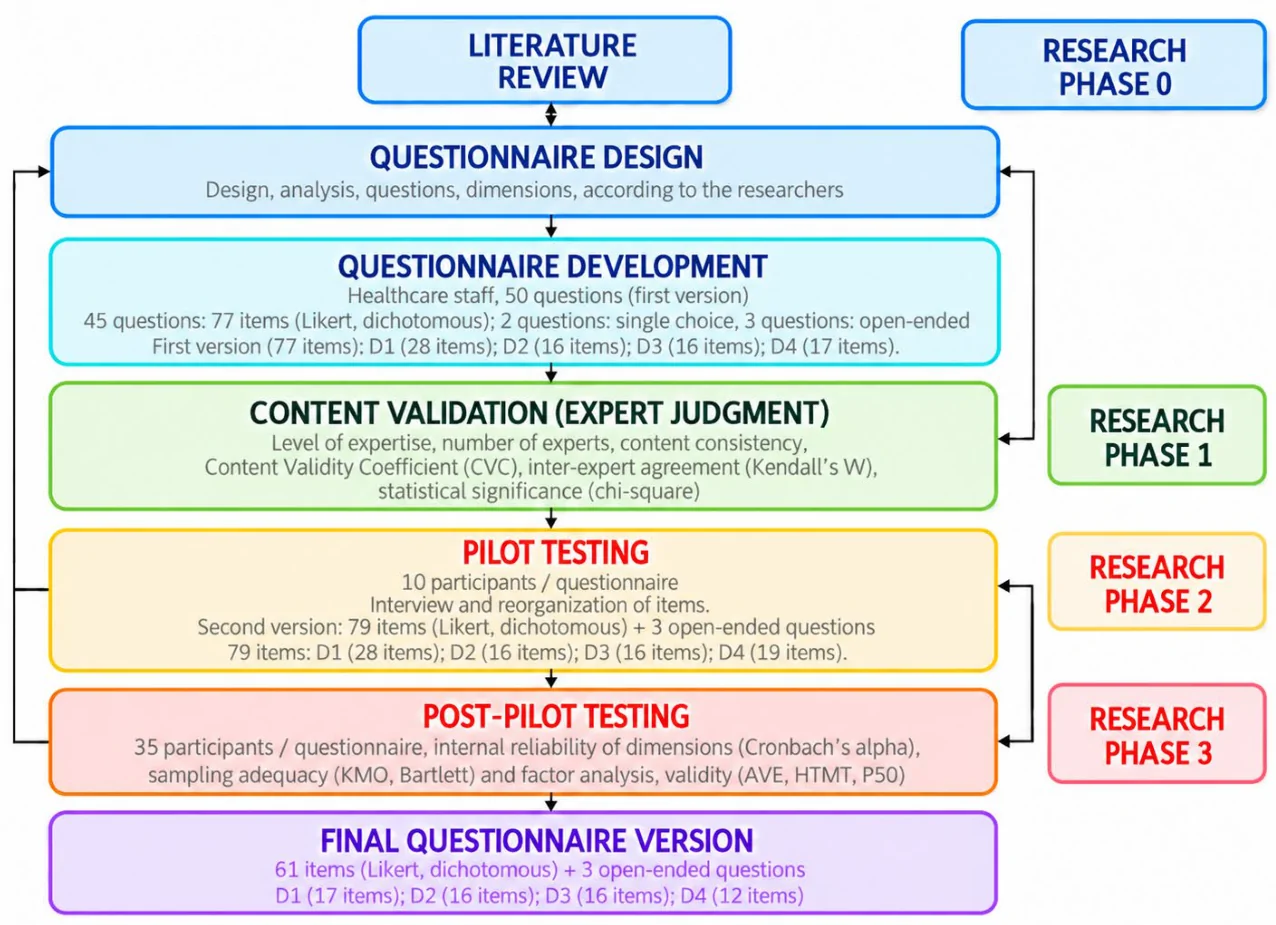
General Study Outline.

The questionnaire was developed and preliminarily evaluated through a four-phase methodological process (Figure 1), following established recommendations for questionnaire development and preliminary psychometric evaluation [31,32]. The term “Phase 0” denotes the initial design stage preceding the empirical content-validation and participant-testing phases.

### 2.1. Phase 0: Questionnaire Development

The instrument was developed based on a literature review addressing healthcare quality, patient safety, the rights of hospitalized children, professional ethics, and the humanization of care [33,34,35,36,37,38,39]. The initial item pool was developed by GRL and JJCA, both of whom have extensive relevant clinical or methodological experience. GRL contributed clinical expertise in pediatric hospital care and children’s rights, whereas JJCA contributed expertise in public health, healthcare quality, research methodology, and questionnaire development. Neither investigator participated as a member of the eight-expert content-validation panel in Phase 1.

**Figure 2:**
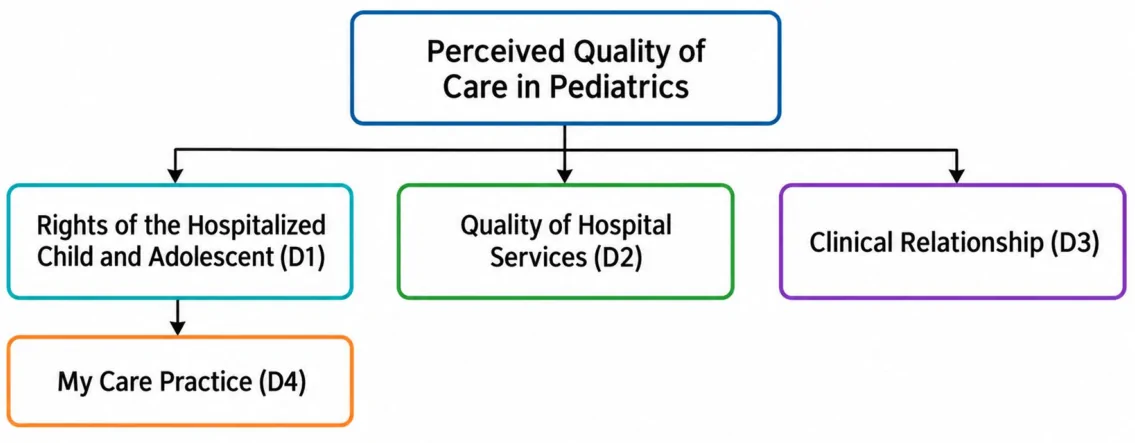
Conceptual framework of the questionnaire.

The initial questionnaire consisted of 50 questions, including 45 questions comprising 77 items (72 Likert-type and 5 dichotomous), 2 single-choice questions, and 3 open-ended questions. The questionnaire incorporated 5-point Likert-type response scales (Strongly Disagree; Disagree; Neither Disagree nor Agree; Agree; Strongly Agree), scored from “1” to “5”, respectively, as well as dichotomous items coded as “Yes/No.” The questionnaire was intended for healthcare professionals working in pediatric hospital care and included sociodemographic variables such as sex, age, profession, and clinical experience. Four theoretical dimensions were defined a priori on the basis of the conceptual framework and the intended content of the instrument: (D1, 28 ítems) Rights of the Hospitalized Child and Adolescent, (D2, 16 ítems) Quality of Hospital Services, (D3, 16 ítems) Clinical Relationship, and (D4, 17 ítems) My Care Practice (Figure 1 and Figure 2). These dimensions were established before participant-based psychometric analyses and were intended to provide the conceptual organization of the questionnaire. Their empirical dimensional structure was not assumed to be established by the present study.

### 2.2. Phase 1: Content Validation

Content validity was assessed through expert judgment involving eight experts: one jurist specializing in family and child law; two clinical psychologists specializing in children and adolescents; one PhD specialist in research methodology; two pediatricians, including one pediatric nephrologist and one specialist in adolescent medicine with a PhD; one physiotherapist with a PhD; and one social worker from community social services. Experts were selected according to predefined competence criteria in health sciences and children’s rights.

The Content Validity Coefficient (CVC), according to the method of Hernández-Nieto [29], and Kendall’s coefficient of concordance (W) were used to assess the adequacy and agreement of expert ratings. Items with a CVC ≥ 0.80 were retained, whereas items showing lower ratings for relevance, appropriateness, or clarity were revised or removed. Comments or justifications from the experts were formally compiled. Each expert independently evaluated every item for relevance, appropriateness, and clarity using a structured rating form. In addition to quantitative ratings, experts could provide free-text comments and suggestions for modification. Individual ratings and qualitative comments were compiled by the research team. No group consensus meeting was conducted. Items were subsequently reviewed by the research team considering both the quantitative CVC results and the qualitative suggestions provided by the experts.

### 2.3. Phase 2: Pilot Testing

The preliminary version of the questionnaire was administered to 10 healthcare professionals to assess item comprehensibility, the appropriateness of the response format, the overall length of the instrument, and any potential difficulties encountered during completion. Participants’ feedback was collected through an interview conducted after completion of the questionnaire.

Based on this feedback, some modifications were made to the instrument. The two “single-choice questions” were converted into “dichotomous” items and incorporated into D4. Consequently, the number of scorable items increased from 77 to 79, distributed across the four a priori theoretical dimensions as follows: D1 (28 items), D2 (16 items), D3 (16 items), and D4 (19 items) (Figure 1).

### 2.4. Phase 3: Preliminary Psychometric Evaluation (Post-Pilot Testing)

Following the pilot phase, the revised questionnaire was administered to 35 healthcare professionals, including pediatricians, Family Medicine residents, and nurses, from the Pediatrics Department of the Nuestra Señora del Prado General University Hospital (SESCAM), Talavera de la Reina, Toledo, Spain, between January and March 2025.

A census sampling approach was used, including all eligible active professionals in the participating department who had not participated in the preliminary pilot phase. Thus, the 35 participants represented the complete population of eligible professionals available for this preliminary evaluation within the participating department. Nevertheless, the absolute sample size was considered insufficient for definitive psychometric validation or for establishing a stable factorial structure.

Internal consistency was assessed for the theoretical dimensions, while item performance, sampling adequacy, and exploratory dimensional patterns were examined. Following the post-pilot item analyses, eighteen items were removed to improve the internal consistency of Dimensions D1 and D4. Therefore, the final version of the questionnaire comprised 61 items: 17 in D1, 16 in D2, 16 in D3, and 12 in D4 (Table S1: Final questionnaire 61 items).

The present study was designed as an instrument-development and preliminary psychometric evaluation study. The four dimensions were specified a priori on theoretical and content grounds and were used to organize item development, expert assessment, item refinement, and preliminary internal-consistency analyses. The post-pilot sample of 35 participants was considered appropriate only for preliminary examination of item performance and internal consistency within the participating department. It was not considered sufficient to establish the latent or factorial structure of the 61-item questionnaire. Accordingly, the principal component analyses and other psychometric indicators reported in this study are presented solely as exploratory and hypothesis-generating analyses. Confirmation of the dimensional structure requires substantially larger and independent samples.

### 2.5. Statistical Analysis

Content validity was assessed using the Content Validity Coefficient (CVC). The number of experts was determined using a sample-size formula for estimating proportions in descriptive studies [40]. Inter-rater agreement was assessed using Kendall’s W coefficient. Corrected item–total correlations were reviewed during pilot refinement, with negative or very low correlations prompting consideration of item removal together with the resulting change in Cronbach’s alpha.

Statistical analyses were performed using IBM SPSS Statistics, version 31.0.1.0. Descriptive analyses included measures of central tendency and dispersion for quantitative variables and frequencies and percentages for categorical variables. Where appropriate, comparisons between groups were performed using Student’s *t*-test, Pearson’s chi-square test, or one-way analysis of variance (ANOVA). Associations between quantitative variables were assessed using Pearson’s or Spearman’s correlation coefficients, as appropriate. For descriptive interpretation of the reported solutions, component loadings and communalities of 0.50 or greater were treated as salient. The number of retained components was based on the eigenvalue >1 criterion; a scree-plot criterion was not used in the reported analysis, which further supports treating these solutions as exploratory.

Internal consistency was estimated using Cronbach’s alpha, with values of α ≥ 0.70 considered acceptable for this preliminary exploratory evaluation. Item analysis included assessment of the percentage of missing responses and examination of potential floor and ceiling effects.

Sampling adequacy for the exploratory component analyses was assessed using the Kaiser–Meyer–Olkin (KMO) measure. KMO values below 0.50 were considered unacceptable. Bartlett’s test of sphericity was used to assess whether the correlation matrix differed significantly from an identity matrix.

Principal component analysis (PCA) was conducted as an exploratory data-reduction procedure. The questionnaire was conceptually developed around four theoretical dimensions specified a priori as domains contributing to the overarching construct of healthcare professionals’ perceived quality of pediatric hospital care. Separate PCAs were therefore conducted within each theoretical dimension to explore patterns of association and empirical item groupings within the respective domains. This approach was not intended to establish or confirm the factorial structure of the overall instrument. Accordingly, the extracted components were interpreted as exploratory empirical groupings of items rather than as confirmed latent factors. Components with eigenvalues greater than 1 were initially retained, followed by Varimax rotation to facilitate the descriptive interpretation of the component solutions. A scree-plot criterion was not used. Component loadings and communalities of 0.50 or greater were considered salient for descriptive purposes.

The decision not to perform a joint factor analysis of the complete item set was based on the limited sample size relative to the number of questionnaire items. Methodological research has demonstrated that sample-size requirements for factor analysis depend on several interrelated characteristics, including communalities, factor overdetermination, the number of factors, and the characteristics of the underlying measurement model, rather than on a fixed participant-to-variable ratio [41,42]. Although satisfactory factor recovery may be achieved with small samples under particularly favorable conditions [43], elevated communalities alone do not guarantee sampling adequacy or the stability of the resulting factor solutions. In the present study, the combination of a limited sample size, a comparatively large item set, an empirically unconfirmed dimensional structure, and modest KMO values did not provide sufficient grounds to assume that a joint factor analysis would yield a stable and reproducible factorial solution.

Furthermore, because the number of items exceeded the number of participants, the sample covariance matrix for the complete item set would necessarily be singular. Although a joint PCA could technically be calculated, obtaining a component solution under these conditions would not, by itself, provide reliable evidence of the questionnaire’s underlying latent structure. Consequently, the separate PCAs were retained exclusively as descriptive, exploratory, and hypothesis-generating analyses. Their results were not considered evidence establishing the empirical dimensional structure of either the individual theoretical dimensions or the questionnaire as a whole.

As secondary exploratory analyses, average variance extracted (AVE) and the heterotrait–monotrait ratio (HTMT) were calculated to provide descriptive information about the relationships among the proposed theoretical dimensions. These analyses were not intended to establish convergent or discriminant validity because of the limited sample size and the provisional nature of the dimensional structure.

The association between questionnaire scores and the global satisfaction item (P50) was examined as an exploratory association. Because P50 was part of the questionnaire rather than an independent external criterion, this analysis was not interpreted as definitive evidence of criterion-related validity.

### 2.6. Ethical and Legal Aspects

The protocol received favorable approval from the Research and Medicines Ethics Committee (CEIm) of the Talavera de la Reina Integrated Care Management (Opinion 25/2024). The evaluated rights correspond to the European Charter for Children in Hospital (European Parliament, 1993). Confidentiality is guaranteed through data protection in a file that complies with current legislation, including Law 41/2002, Law 44/2003, Law 14/2007, Regulation (EU) 2016/679, and Organic Law 3/2018. Written informed consent was obtained from all participants before their participation in the study.

## 3. Results

### 3.1. Content Validation

Content validation of the questionnaire, conducted by eight experts, showed high CVC values for relevance (0.976), appropriateness (0.982), and clarity (0.973). According to the criteria of Hernández-Nieto [29], these values were considered “excellent,” supporting the quality of the items in terms of clarity, relevance, and appropriateness for the construct being assessed.

### 3.2. Questionnaire Reliability

Inter-rater agreement was evaluated using Kendall’s W coefficient, yielding an overall value of W = 0.85, indicating a high and statistically significant level of agreement (*p* < 0.001). The items with the lowest agreement (W = 0.60) were P5, P9, P10, P19, and P25, whereas those with complete agreement (W = 1.00) were P1, P2, P3, P4, P6, P7, and P8. Based on expert feedback, 25 items were revised, with no items requiring elimination at this stage.

### 3.3. Internal Consistency of the Questionnaire (Preliminary Pilot Testing)

In the initial pilot study (*n* = 10), the questionnaire showed a descriptive preliminary estimate Cronbach alpha of 0.596 across 77 items. Their observations led to modifying the 2 “single-choice questions” and converting them into “dichotomous” items. The questionnaire at the end of this phase consisted of 79 items, which was to be administered to healthcare personnel (*n* = 35).

### 3.4. Preliminary Psychometric Evaluation (Post-Pilot Testing)

The questionnaire was administered to 35 professionals, including 12 men (34.3%), whose mean age was 39.1 ± 9.16 years, and 23 women (65.7%), whose mean age was 48.4 ± 10.99 years. Participants included 5 pediatricians (14.3%), 10 residents (28.6%), and 20 nurses (57.1%), with women predominating among nurses (*n* = 16; 80%). Statistically significant associations (*p* < 0.05) were found between age and sex, sex and years of experience, and age and years of experience. No missing values were recorded for the items (Table 1). Each dimension was analyzed separately (D1–D4).

### 3.5. Internal Reliability by Dimensions

We increased the internal reliability of the four dimensions as follows:

- Rights of the hospitalized child and adolescent (D1): A total of 11 items were eliminated to improve internal consistency, moving from α = 0.600 (28 items) to α = 0.733 (17 items), thereby reaching the acceptability threshold.
- Hospital service quality (D2): Adequate internal consistency with α = 0.765 was achieved for 16 items.
- Clinical relationship (D3): Adequate internal consistency with α = 0.718 was achieved for 16 items.
- My care practice (D4): A total of 7 items were eliminated to improve internal consistency, moving from α = 0.596 (19 items) to α = 0.782 (12 items), reaching the acceptability threshold (Table 2).

The four theoretical dimensions were prespecified during questionnaire development based on the conceptual framework used to define the overarching construct. The separate principal component analyses were conducted as exploratory analyses of item groupings within each dimension and were not intended to establish or confirm factors underlying the respective dimensions.

### 3.6. Sampling Adequacy and Exploratory Component Analysis

The sampling adequacy and exploratory component analyses were performed as follows:

- D1 (Rights of the hospitalized child and adolescent): KMO = 0.522; Bartlett’s χ^2^ = 221.05; df = 136; *p* < 0.001. Six components were retained according to the eigenvalue >1 criterion, accounting for 72.1% of the variance. Following Varimax rotation, the items were distributed across six components. Given the small sample size and limited sampling adequacy, this solution should be regarded as exploratory and hypothesis-generating rather than as evidence of an empirically established dimensional structure.
- D2 (Quality of Hospital Services): The exploratory component analysis provided preliminary information on item relationships within this a priori theoretical dimension. Most items showed communalities and component loadings above 0.60. These findings are descriptive and exploratory and should not be interpreted as confirmation of the proposed dimensional structure. Additional exploratory indicators, including AVE, HTMT, and the association with the global satisfaction item (P50), are presented separately below and were not considered definitive evidence of construct validity (Table 3).
- D3 (Clinical relationship): KMO = 0.545; Bartlett’s χ^2^ = 253.59; df = 120; *p* < 0.001. Six components were retained according to the eigenvalue >1 criterion, accounting for 76.5% of the variance. Following Varimax rotation, the items were distributed across six components. Given the small sample size and limited sampling adequacy, this solution should be regarded as exploratory and hypothesis-generating rather than as evidence of an empirically established dimensional structure.
- D4 (My care practice): KMO = 0.531; Bartlett’s χ^2^ = 129.37; df = 66; *p* < 0.001. Four components were retained according to the eigenvalue >1 criterion, accounting for 65.9% of the variance. Following Varimax rotation, the items were distributed across four components. Given the small sample size and limited sampling adequacy, this solution should be regarded as exploratory and hypothesis-generating rather than as evidence of an empirically established dimensional structure (Table 2).

### 3.7. Exploratory Psychometric Findings

The exploratory component analyses and additional psychometric indicators provided preliminary information on item behavior and relationships among the theoretically defined dimensions. Communalities and component loadings were examined descriptively, while AVE and HTMT were calculated as exploratory indicators of the relationships among the proposed dimensions. Given the small sample size, modest KMO values, and provisional nature of the component structure, these analyses should not be interpreted as definitive evidence of convergent or discriminant validity. Similarly, the association with the global satisfaction item (P50), including its loading onto D4 component 2 (communality = 0.60), was examined as an exploratory association rather than as evidence of criterion-related validity because P50 forms part of the questionnaire and is not an independent external criterion (Table 3).

## 4. Discussion

This study describes the development and preliminary psychometric evaluation of a questionnaire designed to assess four complementary a priori theoretical dimensions of pediatric hospital care: Rights of the Hospitalized Child and Adolescent (D1), Quality of Hospital Services (D2), Clinical Relationship (D3), and My Care Practice (D4). The instrument was developed through a structured methodological process incorporating literature and regulatory review, expert content assessment, pilot testing, item refinement, and preliminary psychometric analysis, in accordance with recommendations for questionnaire development and psychometric evaluation in health sciences [29,31,32,44]. During Phase 0, no single psychometrically evaluated instrument was identified that comprehensively integrated these four domains within the specific context of pediatric hospital care. Existing instruments and frameworks address relevant aspects of pediatric healthcare, including quality of care, hospitalized children’s rights, and patient and family experience, but generally focus on more specific domains [16,26,27,28]. This provided the conceptual rationale for developing an instrument capable of integrating these complementary aspects of pediatric hospital care within a single framework.

The instrument underwent progressive refinement throughout the development process. Following pilot testing, the two pre-existing single-choice questions, which were not included in the initial 77-item dimensional count, were reformatted as dichotomous items and incorporated into D4, increasing the dimensional item set to 79 items. Subsequent post-pilot refinement led to the removal of 18 items, resulting in a final 61-item questionnaire distributed across D1 (17 items), D2 (16 items), D3 (16 items), and D4 (12 items).

The content-validation phase yielded consistently high coefficients. Content Validity Coefficient values were 0.976 for relevance, 0.982 for appropriateness, and 0.973 for clarity, while Kendall’s coefficient of concordance (W = 0.85, *p* < 0.001) indicated a high degree of agreement among the eight experts. These findings support the adequacy and representativeness of the items in relation to the intended content domains. Establishing a clearly defined conceptual framework and combining structured expert judgment with quantitative content-validity indicators are fundamental steps in questionnaire development, particularly for instruments encompassing multiple related domains [31,45,46]. Nevertheless, content validity should be distinguished from structural validity: agreement among experts regarding item relevance and representativeness does not, by itself, demonstrate that the proposed theoretical dimensions correspond to empirically distinct latent constructs.

An additional mathematical limitation arises from the relationship between sample size and the number of scored items [47]. With 61 dimensional items and 35 participants, the complete sample covariance or correlation matrix is necessarily rank-deficient. Although a joint PCA could technically be computed, the resulting component solution would remain strongly constrained by the available sample and would not, by itself, provide reliable evidence regarding the questionnaire’s latent structure. Replacing PCA with another exploratory factor-analytic procedure using the same dataset would therefore not overcome the fundamental limitations imposed by the sample. For this reason, the dimension-specific PCAs were retained exclusively as descriptive, exploratory, and hypothesis-generating analyses.

The separate analysis of the four prespecified theoretical dimensions also has an important interpretative implication. Examining item groupings separately within D1–D4 can provide descriptive information about patterns of association among items assigned to each theoretical domain, but it cannot establish whether the four dimensions are empirically distinct from one another, whether each dimension is unidimensional, or whether the complete item set adequately represents an overarching latent construct. Consequently, the four dimensions should continue to be regarded as a priori theoretical domains derived from the conceptual framework and intended content of the questionnaire rather than as empirically confirmed latent factors. Their dimensional organization remains to be evaluated using appropriately powered exploratory and/or confirmatory measurement models in independent validation samples [29,41,42,43,48].

AVE and HTMT should be interpreted within the same exploratory framework. In the present study, these indices were calculated as secondary descriptive indicators of relationships among the proposed theoretical dimensions. However, because the dimensional structure has not yet been independently established and the available sample is limited, these results should not be interpreted as definitive evidence of convergent or discriminant validity. Formal evaluation of these properties requires an adequately specified measurement model examined in a substantially larger and independent sample. Thus, the present AVE and HTMT findings are best regarded as exploratory and hypothesis-generating rather than confirmatory.

The analysis involving the global satisfaction item P50 requires similar caution. P50 showed an exploratory association with questionnaire scores and loaded on the second component identified within D4, with a communality of 0.60. These findings may indicate a relationship between global satisfaction and aspects of perceived pediatric hospital care. However, P50 forms part of the questionnaire itself and therefore does not constitute an independent external criterion. Its associations with questionnaire scores cannot consequently be considered definitive evidence of criterion-related validity. Future validation studies should evaluate relationships with independent external measures or previously validated instruments assessing conceptually related constructs.

Several limitations should be considered when interpreting these findings. The principal limitation is the small post-pilot sample (*n* = 35) relative to the number of questionnaire items. Although these participants represented the complete population of eligible healthcare professionals available in the participating Pediatrics Department who had not participated in the preliminary pilot phase, the absolute sample size remains insufficient to establish a stable empirical dimensional structure. The modest KMO values further reinforce the need for cautious interpretation. In addition, item refinement and estimation of internal consistency were performed using the same post-pilot sample, creating the possibility that the resulting coefficients partly reflect sample-specific relationships. The component solutions and internal-consistency estimates therefore require independent replication.

A further limitation is the single-center design. The study was conducted within one pediatric department, potentially limiting the generalizability of the findings to other healthcare professionals, pediatric settings, institutions, and organizational contexts. Moreover, because the dimension-specific PCAs were exploratory and the complete dimensional structure was not evaluated jointly, the present study cannot determine whether the proposed four-domain conceptual framework corresponds to the empirical latent organization of the instrument. Similarly, the exploratory AVE, HTMT, and P50 findings cannot substitute for formal evidence of convergent, discriminant, or criterion-related validity.

These limitations define the level of psychometric evidence that can reasonably be derived from the present study but do not negate the value of the instrument-development process. The strongest evidence obtained concerns the conceptual development of the questionnaire, expert-based content validity, pilot-informed item refinement, and preliminary internal consistency within the participating sample. In contrast, PCA, AVE, HTMT, and the associations involving P50 provide secondary exploratory information and should not be regarded as confirmation of structural, convergent, discriminant, or criterion-related validity. This distinction is essential to avoid interpreting an initial psychometric evaluation as a completed validation process.

Future validation studies should recruit substantially larger and independent samples, preferably from multiple pediatric healthcare settings. The complete dimensional item set should be evaluated jointly using appropriately powered exploratory and/or confirmatory factor-analytic procedures selected according to the hypothesized measurement model and the characteristics of the item distributions. These studies should determine whether the four proposed theoretical dimensions correspond to empirically supported latent factors, reassess item performance and internal consistency using complementary coefficients such as McDonald’s omega [49], evaluate test–retest reliability, and examine associations with independent external criteria. Where sample size permits, measurement invariance across relevant professional or institutional groups should also be investigated.

Taken together, the present study represents an initial stage in the psychometric development of the questionnaire rather than a completed validation process. The findings support its conceptual development and expert-assessed content validity and provide preliminary evidence of internal consistency within the participating sample. The exploratory component analyses offer initial information regarding item behavior and empirical patterns of association within the four proposed theoretical dimensions but do not establish their latent structure. Confirmation of dimensionality, reliability, and broader validity will therefore require replication in substantially larger and independent validation cohorts.

## 5. Conclusions

This study describes the structured development and preliminary psychometric evaluation of a questionnaire designed to assess complementary aspects of comprehensive protection, quality, and humanization in pediatric hospital care. Its principal contribution lies in the systematic development of the instrument, expert-based content assessment, pilot-guided item refinement, and preliminary evaluation of internal consistency, resulting in a final 61-item questionnaire organized into four a priori theoretical dimensions.

The findings provide strong expert-based evidence of content validity and preliminary evidence of acceptable internal consistency across the four theoretical dimensions. However, these dimensions represent the conceptual organization of the instrument and should not yet be interpreted as empirically confirmed latent factors. The dimension-specific principal component analyses provided exploratory and descriptive information on item relationships within the proposed domains but did not establish structural validity or confirm the latent dimensional structure of the questionnaire.

Accordingly, the present findings should be regarded as an initial stage in the psychometric development of the instrument rather than as evidence of completed validation. The internal-consistency estimates also require independent replication. Further studies using substantially larger, independent, and preferably multicenter samples are needed to evaluate the proposed dimensional structure and additional measurement properties, including complementary reliability estimates such as McDonald’s omega, test–retest reliability, convergent and discriminant validity within an appropriate measurement model, and criterion-related validity using independent external criteria.

Subject to further psychometric confirmation, the questionnaire may have potential utility for assessing healthcare professionals’ perceptions of comprehensive protection, quality, and humanization in pediatric hospital care and may contribute to research, professional training, quality-improvement strategies, and humanization initiatives.

## Figures and Tables

**Table 1:** Sociodemographic data of the participants (*n*:35).

Profession	Age $\overset{-}{x}$ Years ± SD	Experience $\overset{-}{x}$ Years ± SD	Sex M (%)	Sex F (%)
Pediatricians	46.0 ± 10.42	18.4 ± 12.54	3 (8.6)	2 (5.7)
Residents	35.7 ± 5.89	8.8 ± 6.61	5 (14.3)	5 (14.3)
Nursing	49.8 ± 10.75	23.0 ± 7.12	4 (11.4)	16 (45.7)
Total (n:35)	45.23 ± 11.21	18.29 ± 9.90	12 (34.3)	23 (65.7)

SD: standard deviation.

**Table 2:** Comparison of Questionnaire Dimensions (final version: 61 items).

Dimension	Items	Items by Dimension	Cronbach’s Alpha	KMO	Bartlett (χ^2^; df; *p*)	Extracted Factors	Variance Explained by Factor	Communalities (Range)
D1	17	P1, P3, P8, P8.a, P10, P11, P14, P16.1, P16.2, P16.3, P16.4, P16.6, P16.7, P16.8, P16.9, P16.10, P17	0.733	0.522	221; 05; 136; <0.001	6	F1 = 15.1% F2 = 13.4% F3 = 13.1% F4 = 13.0% F5 = 9.4% F6 = 8.0%	0.65–0.82
D2	16	P19, P20, P21.1, P21.2, P21.3, P21.4, P21.5, P21.6, P21.7, P21.8, P22, P23, P24, P25, P26, P27	0.765	0.515	233; 63; 120; <0.001	5	F1 = 19.0% F2 = 16.8% F3 = 13.3% F4 = 11.0% F5 = 9.4%	0.56–0.84
D3	16	P28.1, P28.2, P28.3, P28.4, P28.5, P28.6, P30, P31, P32, P33, P34, P35, P36, P37, P38, P39	0.718	0.545	253; 59; 120; <0.001	6	F1 = 19.0% F2 = 16.4% F3 = 13.5% F4 = 11.6% F5 = 8.3% F6 = 7.7%	0.50–0.87
D4	12	P40.2, P40.3, P40.6, P40.7, P40.8, P40.9, P40.10, P41, P42, P43, P45, P50	0.782	0.531	129; 37; 66; <0.001	4	F1 = 21.1% F2 = 18.8% F3 = 14.9% F4 = 11.1%	0.53–0.83

Source. Own elaboration.

**Table 3:** Comparative Dimensions of the Questionnaire (final version: 61 items).

Dimension	Items	Items with Highest Load	Problematic Items	Convergent Validity	Discriminant Validity	Criterion Validity
D1	17	P16.2 (0.842), P8.a (0.841), P14 (0.839), P11 (0.822), P17 (0.809)	None critical	Communalities and loads > 0.6	6 differentiated factors	Positive correlation with P50
D2	16	P21.3 (0.844), P21.6 (0.802), P25 (0.886), P24 (0.768)	P21.5 (0.557)	Communalities and loads > 0.60 in most items	5 differentiated factors	Positive correlation with P50
D3	16	P31 (0.910), P28.6 (0.898), P28.2 (0.919), P35 (0.884), P34 (0.863)	P33 (0.505)	Communalities and loads > 0.60 in most items	6 differentiated factors	Positive correlation with P50
D4	12	P40.8 (0.899), P45 (0.799), P43 (0.760), P50 (0.757), P40.6 (0.796)	P42 (−0.663)	Communalities and loads ≥ 0.50 in most items	4 differentiated factors	P50 saturation on F2 (communality = 0.60)

Source. Own elaboration.

## Data Availability

The original contributions presented in this study are included in the article/Supplementary Material. Further inquiries can be directed to the corresponding author.

## Supplementary Materials

The following supporting information can be downloaded at https://www.mdpi.com/article/10.3390/pediatric18050137/s1. Table S1: Final questionnaire 61 items.
