# Supplementary material for: Development and Preliminary Psychometric Evaluation of a Questionnaire on Comprehensive Protection, Quality, and Humanization in Pediatric Hospital Care
Source: Pediatr Rep. 2026 Oct 12;18(5):137. doi: 10.3390/pediatric18050137 (PMC13708048; doi:10.3390/pediatric18050137)
Supplement: Supplementary file 1 [file pediatrrep-18-00137-s001.zip › Supplementary Table S1_Final questionnaire 61 items.pdf]

ITEM	QUESTION	L (Likert)<br>D (Dichotomous)
**D1**	**RIGHTS OF THE HOSPITALIZED CHILD AND ADOLESCENT**	
P1	Are you familiar with the European Charter (1986) outlining the 23 Rights of the Hospitalized Child?	D
P3	Promoting the Rights of the Hospitalized Child within the hospital environment is essential.	L
P8	11-year-old children already have the legal capacity to give their opinion on their health status, and I must take that opinion into account.	L
P8.a	If you disagree, at what age does the Law grant them that right?	D
P10	I explain to all children and adolescents the procedures and/or medical acts I am going to perform on them.	L
P11	I know what informed assent is.	D
P14	The hospital institution is responsible for ensuring that the physical, safety, emotional, social, and cognitive needs of the child and adolescent are met during their hospitalization.	L
P16.1	During the child or adolescent's hospitalization, allowing them to play is 'more of a right than a need'.	L
P16.2	"During the child or adolescent's hospitalization, sleeping well is 'more of a right than a need'.	L
P16.3	During the child or adolescent's hospitalization, being able to express what they dislike is 'more of a right than a need'.	L
P16.4	During the child or adolescent's hospitalization, receiving good treatment is 'more of a right than a need'.	L
P16.6	During the child or adolescent's hospitalization, having their opinion on their health heard is 'more of a right than a need'.	L
P16.7	During the child or adolescent's hospitalization, receiving their parents' affection is 'more of a right than a need'	L
P16.8	During the child or adolescent's hospitalization, being informed of their behavioral limits is 'more of a right than a need'.	L
P16.9	During the child or adolescent's hospitalization, being able to interact with other hospitalized children is 'more of a right than a need'.	L
P16.10	During the child or adolescent's hospitalization, being informed of safety rules is 'more of a right than a need'.	L
P17	I recognize that the needs and rights of hospitalized children and adolescents share the principles of generality, obligatoriness, and universality.	L

ITEM	QUESTION	L (Likert)<br>D (Dichotomous)
**D2**	**HOSPITAL SERVICE QUALITY**	
P19	In the emergency department of a hospital, the continuous 24/7 presence of healthcare personnel trained and skilled in the care of pediatric patients is important.	L
P20	Auxiliary service spaces (clinical laboratory, radiology) in a hospital should be child- and adolescent-friendly for hospitalized patients.	L
P21.1	In my view, the characteristics of a dedicated pediatric emergency department include: Reducing patient waiting times.	L
P21.2	In my view, the characteristics of a dedicated pediatric emergency department include: Decreasing the number of unnecessary hospitalizations.	L
P21.3	In my view, the characteristics of a dedicated pediatric emergency department include: Decreasing the performance of diagnostic tests (lab and X-ray).	L
P21.4	In my view, the characteristics of a dedicated pediatric emergency department include: Adding 'value' to the hospital institution.	L
P21.5	In my view, the characteristics of a dedicated pediatric emergency department include: Improving the quality of care for children and adolescents.	L
P21.6	In my view, the characteristics of a dedicated pediatric emergency department include: Being directly related to their rights.	L
P21.7	In my view, the characteristics of a dedicated pediatric emergency department include: Increasing behaviors related to 'do not do'.	L
P21.8	In my view, the characteristics of a dedicated pediatric emergency department include: Improving triage quality.	L
P22	The design and adaptation of the physical environment in a pediatric inpatient unit are not related to a culture of humanization.	L
P23	I consider that, during hospitalization, the comfort of children and their parents is related solely to the quality of care.	L
P24	I recognize that I am the one who must decide on the optimal treatment or procedure for my hospitalized patient.	L
P25	The primary objective of properly equipping a pediatric ward is to: comply with hospital quality and safety certification.	L
P26	I consider that the food prepared for hospitalized children and adolescents should correspond to their age, preferences, and underlying pathology.	L
P27	In my experience, parents cooperate in maintaining the cleanliness of the rooms and bathrooms during their children's hospitalization.	L

ITEM	QUESTION	L (Likert)<br>D (Dichotomous)
**D3**	**CLINICAL RELATIONSHIP**	
P28.1	In your opinion: categorize the following adverse events that occur in pediatric healthcare: Diagnostic error.	L
P28.2	In your opinion: categorize the following adverse events that occur in pediatric healthcare: Patient fall.	L
P28.3	In your opinion: categorize the following adverse events that occur in pediatric healthcare: Phlebitis at venipuncture sites.	L
P28.4	In your opinion: categorize the following adverse events that occur in pediatric healthcare: Medication error.	L
P28.5	In your opinion: categorize the following adverse events that occur in pediatric healthcare: Healthcare-associated infections.	L
P28.6	In your opinion: categorize the following adverse events that occur in pediatric healthcare: Adverse drug event.	L
P30	In my view, emotional support meetings should be conducted in pediatric inpatient wards for mothers and fathers accompanying their hospitalized children.	L
P31	I consider that educational interventions on breastfeeding, healthy habits, and nutrition should be conducted in pediatric inpatient wards for mothers and fathers.	L
P32	In my view, upon discharge, it is necessary to ask parents about their satisfaction with the healthcare service received at the hospital.	L
P33	Addressing the pediatric or adolescent patient by their name is a good practice.	L
P34	When communicating with the patient or their family members, eye contact must always be maintained.	L
P35	I recognize that the family plays a very important role in planning the care of the hospitalized child or adolescent.	L
P36	When addressing the health condition of a child or adolescent and their parents, I must synchronize my emotions and express them through non-verbal language.	L
P37	The child or adolescent should be informed about their illness according to their age, in addition to listening to them and answering their questions.	L
P38	Authorization from parents or legal guardians must always be requested prior to executing the treatment plan.	L
P39	Achieving a high level of satisfaction among hospitalized children and their parents regarding the service provided by healthcare personnel is essential.	L

ITEM	QUESTION	L (Likert)<br>D (Dichotomous)
**D4**	**MY CLINICAL PRACTICE**	
P40.2	Indicate your degree of agreement with the following characteristics of clinical practice: That it improves through trial and error.	L
P40.3	Indicate your degree of agreement with the following characteristics of clinical practice: That it takes place in a consented manner.	L
P40.6	Indicate your degree of agreement with the following characteristics of clinical practice: That it focuses on the disease.	L
P40.7	Indicate your degree of agreement with the following characteristics of clinical practice: That it is based solely on evidence.	L
P40.8	Indicate your degree of agreement with the following characteristics of clinical practice: That it is safe for the patient.	L
P40.9	Indicate your degree of agreement with the following characteristics of clinical practice: That it allows for improvement over time.	L
P40.10	Indicate your degree of agreement with the following characteristics of clinical practice: That it is patient-centered.	L
P41	How do you evaluate the progression of mood at the 'start' and 'end' of the daily shift for an average healthcare professional in present-day practice?	L
P42	I recognize that it is difficult to control emotions during a hospital work shift.	L
P43	In my view, the average healthcare staff member is satisfied with the workload-to-quality-of-life ratio.	L
P45	In my view, communication between hospital management and its healthcare staff is essential.	L
P50	In my view, healthcare staff in general are satisfied with the work of their hospital institutions.	L
